# Small Obstacle Avoidance Sensor

**DOI:** 10.1155/2013/604538

**Published:** 2013-06-25

**Authors:** Richard H. Vollmerhausen

**Affiliations:** RHV Consulting, LLC, 760 Jacktown Road, Lexington, VA 24450, USA

## Abstract

This paper describes a laser ranging sensor that is suitable for applications like small unmanned aerial vehicles. The hardware consists of a diode emitter array and line-scan charge coupled devices. A structured-light technique measures ranges up to 30 meters for 64 field angles in a 90 degree field of view. Operation is eye safe, and the laser wavelength is not visible to night vision goggles. This paper describes a specific sensor design in order to illustrate performance for a given package size.

## 1. Introduction

The laser ranging sensor (LRS) provides range measurements to multiple points within a wide field of view (FOV) using a structured-light technique. Many forms of structured-light ranging have been proposed; the wide variety of implementations is described elsewhere [1–3]. Structured-light techniques use geometry to determine range; they do not require the high speed circuitry associated with time-of-flight ranging. The LRS described here has the small size and low weight attributes needed for integration onto a small unmanned aerial vehicle.

The LRS concept differs from previous work in three ways. First, emitter to detector offset is used to determine range in the traditional manner. However, an additional orthogonal offset is used to tailor detector signal amplitude versus range. Suppressing short range returns permits operation in dust, fog, and smoke that would otherwise blind the sensor. Suppressing short range returns also reduces the dynamic range handled by the electronics. Second, the current technique permits ranging to multiple field points simultaneously without extensive processing. Third, this paper describes the details of a small, lightweight design that provides tens of meters ranging over a wide FOV with a rapid update rate.

This paper describes a specific LRS design; see Table 1. The physical operating principles are not obscure and do not require an extensive explanation. However, it is difficult to establish realistic performance expectations for a given LRS size and weight. Describing a specific sensor design provides a marker in the design trade space.

Section 2 explains general concepts; details are provided in Sections 3 through 5.  Section 3 describes LRS hardware and describes how the effects of optical distortion and thermal expansion are mitigated.  Section 4 discusses eye safety.  Section 5 provides range performance estimates under various lighting conditions. Discussion and summary are in Section 6.

## 2. Operating Principles

**Figure 1:**
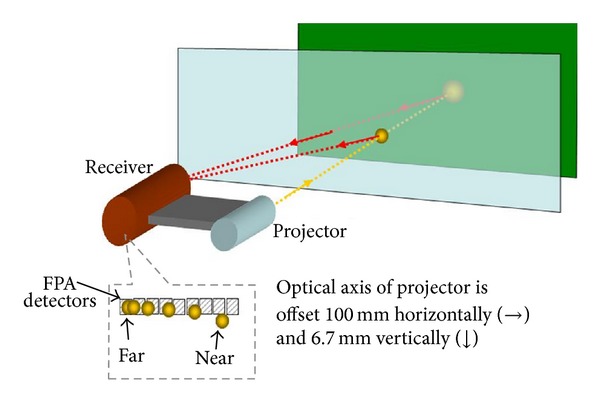
LRS concept.

Figure 1 illustrates the system concept. An optical projector is offset 100 mm to the right (horizontally) and 6.7 mm down (vertically) from an optical receiver. A beam of light emitted by the projector is reflected off a distant object. The angle of arrival of the light at the receiver depends on the distance to the reflecting object. Therefore, the focal plane position of the reflected laser spot depends on object distance. A focal plane array (FPA) is placed in the image plane of the receiver. As illustrated in the callout at the bottom of the figure, the position of the reflected light on the FPA depends on the distance to the object that reflected the light.

Range is determined by the following sequence of events. A pulse time is selected based on eye safety and performance requirements. Background intensities are found by integrating the FPA for a pulse time without the projector emitting light. After reading out background intensities, the FPA integrates for a pulse time with the projector emitting light. Background intensities are subtracted from the second set of pixel intensities. The difference intensities are interpolated to find the FPA position of the laser spot. Object range is a known function of laser spot position.

The small vertical offset provides multiple advantages. As illustrated in the callout at the bottom of Figure 1, near-range returns do not fall directly on a detector. This greatly reduces the dynamic range required of the FPA. Further, without the vertical offset, even a small concentration of airborne particulates could blind the receiver. The vertical offset is used to suppress the near-range signal without affecting the weak signals from distant objects.

**Figure 2:**
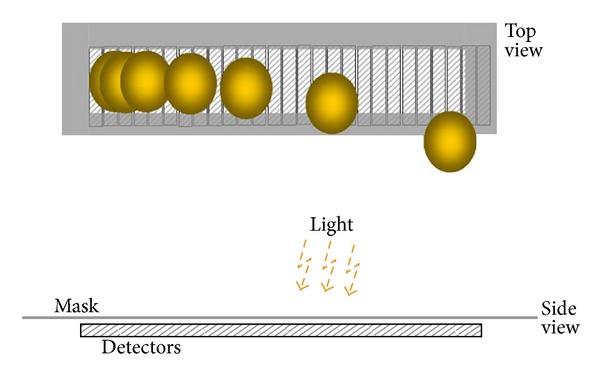
A mask limits the detector area exposed to light.

In Figure 2, the vertical dimension of the FPA detectors is increased, and the extra area is covered by a mask. The larger detectors and mask are needed to account for optical distortion and thermal expansion. Sections 3.2 and 3.3 provide more details.

## 3. Laser Ranging Sensor Hardware

The design objective is 20 meters range performance from a light, compact package. To that end, all lenses and lens separation structures are fabricated from the same polycarbonate material. A single refractive index can be used because of the narrow spectral band of the laser light. The designs here assume that the material is cyclic olefin copolymer (COC), but other choices are available [4]. Fabricating the support structure from polycarbonate material with the same thermal expansion coefficient as the lenses obviates the need for a focus mechanism. 

Plastic has a very low heat capacity, and differential heating can cause both focus and alignment problems. It might be necessary to use a thin metal enclosure or some other mechanism to uniformly distribute heat.

### 3.1. Projector Optics

**Figure 3:**
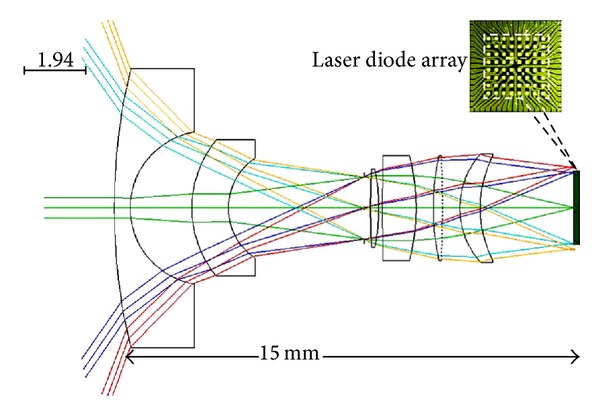
Optical layout of the projector.

The projector optical layout is shown in Figure 3. Sixty-four light beams are projected in a 90 by 90 degree square pattern. Beams at the extreme of each diagonal exit at about 60 degrees from the optical axis. The 2 by 2 millimeter (mm) addressable laser diode array is from Princeton Optronics [5]. The vertical-cavity surface-emitting laser (VCSEL) array has 64 diodes operating at 975 nanometers (nm). The diodes emit in a 20 degree cone defined by the 1/*e* ^2^ intensity. Multimode diodes are selected for increased power and because multimode decreases speckle [6]. Each diode produces 12 milliwatts (mW) of power with 45% conversion efficiency. 

**Figure 4:**
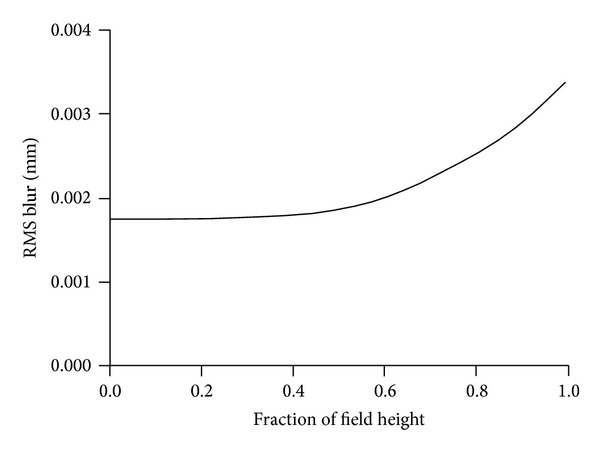
RMS spot diameter versus field height in mm for projector.

All of the lenses are COC.  Figure 4 shows the root of the mean of the squares (RMS) of blur radius versus field height. Dimensions are in millimeters (mm). Note that, in all of the figures, field angle originates from the focal plane and is not the object space field angle.

### 3.2. Receiver Optics

**Figure 5:**
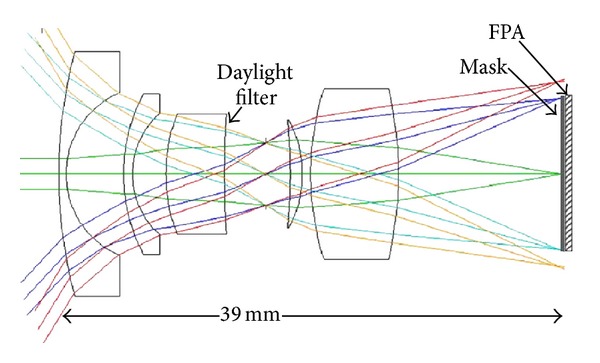
Optical layout of receiver.

**Figure 6:**
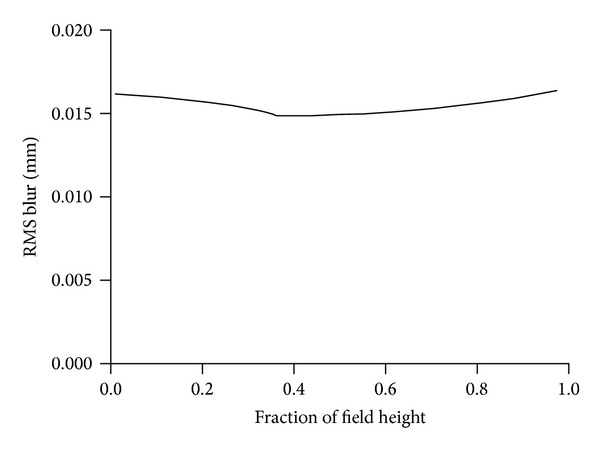
RMS spot diameter versus field height in mm for receiver.

The optical layout of the receiver is shown in Figure 5. All of the lenses are COC.  Figure 6 shows the RMS blur radius versus field height. The optical aperture of the receiver is 3.1 mm^2^, and the numerical aperture (NA) is 0.13. 

Eight linear CCD arrays are situated in the receiver's focal plane. In this example, the arrays are Hamamatsu S9037-0902 512 by 4 back-thinned image sensors [7]. Pixel size is 24 micrometers (*μ*), and so with vertical binning, effective pixel size is 24 by 96 *μ*. The maximum line rate is 15,000 Hertz (Hz). Read noise is 100 electrons per pixel, and dark noise is typically 100 electrons per pixel per second.

The silicon FPAs used in the receiver have a much smaller linear thermal expansion coefficient than polycarbonates [4, 8]. For that reason, the design uses multiple, linear silicon arrays mounted on a polycarbonate substrate. The polycarbonate substrate expands and contracts in the same proportion as the focal length of the receiver, and that maintains the correct distance between the detector arrays. 

Further, the silicon FPAs are overlaid by a reticle printed on polycarbonate. An optical mask is applied to a COC plate and situated just above the FPA. The mask prevents light from reaching the CCD arrays except through selected openings.

Optical distortion changes the position of the laser spots on the focal plane. Also, for laser beams near the edge of the FOV, the spot position follows a slightly curved path as range changes. Blocking most of the laser spot from nearby returns requires the precise position control provided by the mask. Further, laser spot position changes because of thermal expansion. The silicon FPA expands and contracts very little along the length of the array. This means that the group of laser spots shown in Figure 2 moves to different horizontal positions depending on temperature. Since both the mask and optics are polycarbonate, the laser spots and mask opening move in unison.

Operating at 975 nm enhances performance even though FPA quantum efficiency (QE) is low. The QE of the CCD is 0.3 at 975 nm. Using 700 nm laser light would increase the QE to 0.9. However, the laser energy permitted by Class 1 eye safety is 3.55 bigger at 975 nm than at 700 nm [9]. Also, background flux from sunlight increases by a factor of 1.4 at 700 nm. Operating at 975 nm provides better range performance. Also, the laser light is not visible to even the newest generation of night vision goggles [10].

### 3.3. System Characteristics

**Figure 7:**
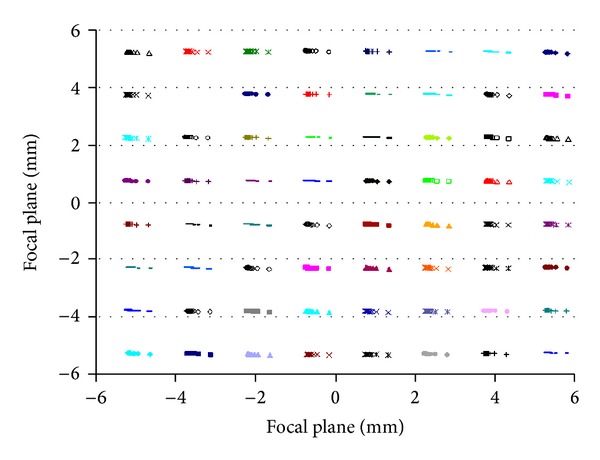
Spot pattern for all 64 laser diodes. Each pattern has 20 points corresponding to 1 to 20 meters range at one meter increment.

**Figure 8:**
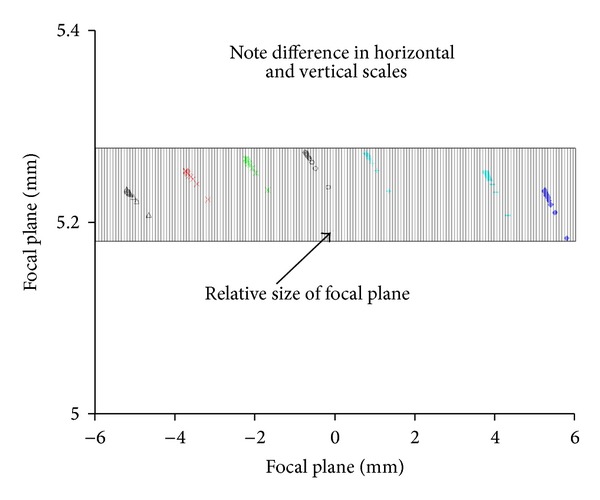
Upper spot patterns from Figure 7. Note the curvature due to distortion. The curvature is exaggerated in this figure by the different vertical and horizontal scales.

Figure 7 shows 64 laser spot patterns in the focal plane of the receiver. Each group has 20 symbols corresponding to 1 to 20 meters at a one meter increment. In Figure 8, the effect of distortion is illustrated by expanding the top row of Figure 7. Note that the ordinate is expanded compared to the abscissa, and so the effect of distortion is visually exaggerated. One benefit of using a mask is that each opening can be tailored for distortion. Both the position and shape of each mask opening can be tailored to fit optical characteristics.

The mask is 0.08 mm wide in order to admit all blur energy at long range. For the receiver optics described in Section 3.2, focal plane position (*F*_*p*_) is given by the approximate formula

(1) $$\begin{array}{c} F_{p}\;=\frac{0.006\;\text{offset}}{\text{Range}\;\;\text{in}\;\;\text{meters}}. \end{array}$$

*F*_*p*_ is relative to the long range laser spot position. In the current example, offset equals 100 mm horizontally and 6.7 mm vertically. As range increases, the blur moves slowly in the vertical direction and faster in the horizontal direction.

**Figure 9:**
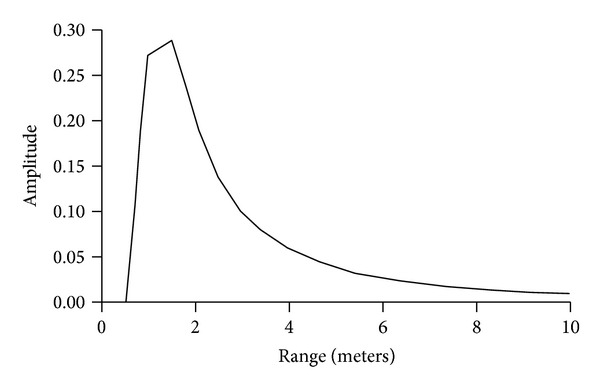
Fraction of transmitted signal on receiver detectors versus range.

Figure 9 shows signal versus range including a range-squared falloff. Returns from nearby objects are suppressed, and signal dynamic range is limited. For a 20 meter range requirement, signal dynamic range is 100 : 1.

## 4. Eye Safety

Eye safety calculations are based on meeting Class 1 according to the international standard [10]. Each individual beam is a fraction of a mm in diameter and almost collimated. Therefore, the maximum power (*P*_max⁡_) from each beam is limited by Table 4 of the standard to

(2) Pmax⁡=(3.9E−4)(100.002(975−700))  watts,Pmax⁡=0.00138  watts.

Two pulse lengths (*t*_*P*_) are used in the range analysis. A *t*_*P*_ of 2.5*E* − 3 seconds is used for room light and daylight. This *t*_*P*_ is too long when the LRS flies directly toward a sunlit wall or sand dune. Direct sunlight overfills the FPA electron wells. Therefore, a *t*_*P*_ of 3*E* − 4 seconds is used for direct sunlit backgrounds.

For 975 nm wavelength, the joules per pulse (*J*_*P*_) is limited to

(3) JP=(7E−4)(100.002(975−700))tP0.75  joules,JP=28E−6  joules  for  2.5E−3  seconds tP,JP=5.7E−6  joules  for  3E−4  seconds tP.

However, the Princeton Optronics laser array used in the current example emits 0.012 watts per diode. The power used for sunlight range calculations is therefore limited to

(4) JP=(0.012)(3E−4),JP=3.6E−6  joules  for  3E−4tP.

When *t*_*P*_ is short, the diode array is not capable of emitting the energy per pulse permitted by the eye safety standard.

The *P*_max⁡_ power restriction limits range update rate to 50 Hz. Total on-time pulse does not further limit the operation. Further, multiple beams can be illuminated simultaneously because each beam is angularly separated by almost 200 milliradians. Retinal damage is the primary hazard when operating at 975 nm. Angular separation spreads the energy from multiple beams to different parts of the retina. For the current example, eye safety is established by the energy and power in a single beam.

## 5. Range Performance

Table 2 summarizes the parameters used in calculations. The background flux is based on a spectral bandwidth of 0.1 *μ*. This wide spectral band is needed because of variation in the filter with temperature and because the input light is not normal to the filter surface.

The sun background assumes that a sunlit surface completely fills the LRS FOV. The background has a reflectivity of 0.5. The daylight background assumes a bright, clear day with the LRS viewing the sky or ground. The room background assumes indoor lighting. In terms of range performance, darker is better. So night performance is better than in room light.

When the LRS views a sunlit scene, the CCD electron well cannot hold all of the photoelectrons generated in 2.5 milliseconds. Therefore, *t*_*P*_ for the sun background is 0.3 milliseconds and not 2.5 milliseconds. For a sunlit background, the signal to background ratio is 1 : 140 for an object at 20 meters range. The signal to noise is high, but good dynamic range is needed from the CCD and electronic processing.

Detector noise (*D*_noi_) is given by (5) and background noise (BCK_noi_) by (6) [11]. The $\sqrt{2}$ factor in (5) is included because the background must be subtracted from the background plus laser signal. Detector signal (*S*_sig_) versus range is given by (7). Consider the following:

(5) $$\begin{array}{c} D_{\text{noi}}=\sqrt{2\left[D_{\text{ark}}t_{P}+R_{\text{noi}}^{2}+\text{BC}{\text{K}}_{\text{noi}}\right],} \end{array}$$

(6) $$\begin{array}{c} \;\;\text{BC}{\text{K}}_{\text{noi}}=\sqrt{\frac{P_{\text{area}}t_{P}B_{\text{flux}}NA^{2}\text{REFB}\;\tau \text{AW}}{q},} \end{array}$$

where *q* is charge on an electron.Consider the following:

(7) $$\begin{array}{c} S_{\text{sig}}=\frac{P_{ix}\text{REFT}A_{p}J_{P}\tau \text{AW}}{\pi R_{\text{ng}}^{2}\;q}, \end{array}$$

where *R*_ng_ is range in mm. The factor *P*_*ix*_ is the fraction of laser spot energy on a detector; the maximum value of *P*_*ix*_ is 0.42.

The CCD is operated twice for each ranging cycle, once with the lasers emitting and once without. The difference signal provides intensities of the laser spots. Linear interpolation is used to find laser spot position on the FPA. Range is found using (1). 

The optical blur is bigger than a CCD pixel, and this degrades interpolation accuracy. At most a fraction 0.42 of the laser energy falls on a single pixel, and most of the remaining energy falls on the two adjacent detectors. Differences in pixel intensities tend to be small, and, at longer ranges, the laser spot does not move very much as range changes. Range accuracy degrades rapidly as range increases.

However, pixel signal to noise is 3.5 : 1 at 35 meters with a sunlit background and 48 meters indoors. The high signal to noise means that laser spot location is known reliably to ±0.5 pixel. Knowing laser spot location to ±0.5 pixel establishes a maximum range error at each range.

**Figure 10:**
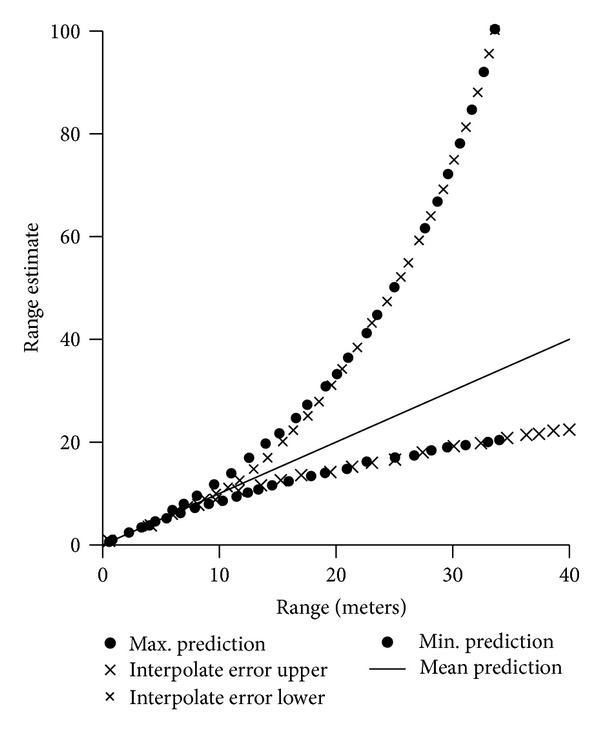
Range error estimates versus range for direct sun. The dashes show maximum and minimum predictions versus range.

**Figure 11:**
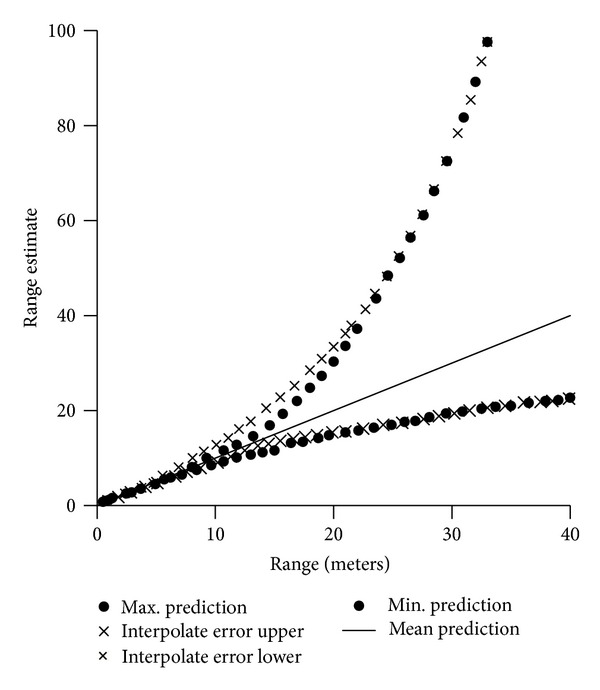
Range error estimates versus range for daylight. The dashes show maximum and minimum predictions versus range.

**Figure 12:**
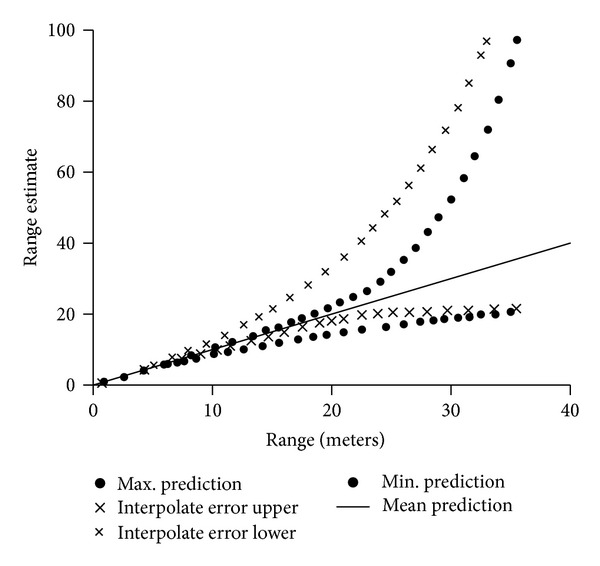
Range error estimates versus range for room light. The dashes show maximum and minimum predictions versus range.

Figures 10, 11, and 12 show range performance for direct sun, daylight, and room light background, respectively. The crosses (×) show the RMS of range error based on interpolation. The dashes (–) show maximum and minimum range predictions based on knowing FPA position to ±0.5 pixel.

## 6. Discussion and Summary

Range error is small at close range and increases at longer range. Small range error at close range is needed to maintain flight path along walls or near the ground. At longer range, the LRS provides sufficient range precision to avoid obstacles and find open flight paths.

Range error scales with the size of the offset between projector and receiver optical axes. Doubling the offset halves the range error, and halving the offset doubles the range error. However, for offsets bigger than 200 mm, the spot patterns in Figure 7 begin to overlap. With the current optical design, range error can be halved by widening the offset.

Achieving longer range performance by increasing laser output power at 975 nm is not practical. Scaling the projector size up by a factor of 10 would not permit any increase in pulse energy. The laser beam would still be smaller than the eye pupil, and the current design already projects the maximum amount of eye-safe energy into the eye pupil.

The primary risk to achieving the range performance shown in Figures 10 through 12 is differential heating. Direct sunlight can mechanically stress the sensor; internal heat generated by electronics also creates stress. Differential heating causes both defocus and misalignment. Risk is mitigated by considering thermal problems when packaging the electronics and when designing the enclosure and cooling surfaces.

The LRS provides the performance needed for confined-space navigation of small aircraft. Fifty times each second, the LRS ranges to 64 field points over a 90 degree FOV. For indoor operation, range accuracy is 2 meters at 20 meters range. This performance is achieved in a small, light package by using a structured-light technique.

Structured-light ranging requires that the projector and receiver remain in focus and well aligned. The LRS accomplishes this by fabricating all of the lenses and mechanical separators out of polycarbonates. The optics stay in focus, and the system stays in alignment over a broad temperature range. Since the silicon FPA does not have the same thermal expansion coefficient as polycarbonates, a polycarbonate mask is used to selectively expose portions of the FPA. The LRS design maintains focus and alignment with sufficient precision that the structured-light technique provides the needed range performance.

## Figures and Tables

**Table 1:** LRS parameters.

Parameter	Value
FOV	90 degree full angle
Angles ranged	64
Range update	50 per second
Receiver volume	10 cubic centimeters
Receiver weight	10 grams
Transmitter volume	0.6 cubic centimeters
Transmitter weight	2 grams

**Table 2:** Parameters used in range calculations. Irradiance values are in watts per square centimeter (watts/cm^2^).

Parameter	Value	Unit	Symbol
Optics transmission	0.8		*τ*
Receiver aperture	3.1	mm^2^	*A*_*p*_
Numerical aperture	0.13		
Object reflectance	0.3		REFT
Background reflectance	0.5		REFB
Background flux (sun)	6*E* − 3	Watts cm^−2^	*B*_flux_
Background flux (daylight)	6*E* − 4	Watts cm^−2^	
Background flux (room)	6*E* − 5	Watts cm^−2^	
Detector responsivity	0.3	Amperes watt^−1^	AW
Detector size	24 by 96	*μ*	
Detector area	2.3*E* − 5	cm^2^	*D*_area_
Read noise	100	Electrons per pixel	*R*_noi_
Dark current	100–1000	Electrons per second	*D*_noi_
Line rate	15000	Lines per second	
Well capacity	600,000	Electrons	
